# Paeoniflorin relieves arterial stiffness induced by a high-fat/high-sugar diet by disrupting the YAP-PPM1B interaction

**DOI:** 10.1093/lifemedi/lnad029

**Published:** 2023-09-12

**Authors:** Zhipeng Chen, Yanan Liu, Mengke Li, Jiawei Song, Jianping Lin, Ding Ai

**Affiliations:** National Key Laboratory of Blood Science, Key Laboratory of Immune Microenvironment and Disease (Ministry of Education), Tianjin Medical University, Tianjin 300070, China; Department of Physiology and Pathophysiology, Tianjin Medical University, Tianjin 300070, China; Department of Physiology and Pathophysiology, Tianjin Medical University, Tianjin 300070, China; Department of Physiology and Pathophysiology, Tianjin Medical University, Tianjin 300070, China; College of Pharmacy, NanKai University, Tianjin 300350, China; Biodesign Center, Tianjin Institute of Industrial Biotechnology, Chinese Academy of Sciences, Tianjin Airport Economic Area, Tianjin 300308, China; National Key Laboratory of Blood Science, Key Laboratory of Immune Microenvironment and Disease (Ministry of Education), Tianjin Medical University, Tianjin 300070, China; Department of Physiology and Pathophysiology, Tianjin Medical University, Tianjin 300070, China

**Dear Editor,**

Arterial stiffness is closely associated with metabolic syndrome, which is a group of cardiovascular risk factors [1, 2]. High-fat/high-sugar (HFHS) diet enhances transforming growth factor (TGF) β-activated Smad2/3 phosphorylation in arteries [3]. Our previous research showed that PPM1B is a yes-associated protein (YAP)-bound phosphatase that is translocated into the nucleus to dephosphorylate Smads in response to TGFβ. However, this process is inhibited by YAP by the removal of the K63-linked ubiquitin chain of protein phosphatase, Mg2+/Mn2+ dependent 1B (PPM1B) at K326 [4]. In the present study, we describe that paeoniflorin, a traditional Chinese medicine, inhibits the development of HFHS-induced arterial stiffness by blocking the YAP-PPM1B interaction. As paeoniflorin plays an anti-tumor role and protects against cardiovascular events [5, 6], this report suggests a new strategy for the clinical treatment of arterial stiffness.

To identify a potential inhibitor of the YAP-PPM1B interaction from a library of 1045 U.S. Food and Drug Administration (FDA)-approved drugs, we used a NanoLuc binary technology high-throughput screening approach, which is based on a two-subunit system for the detection of intracellular protein-protein interactions (Fig. 1A). We fused large BiT with the CC domain of YAP (LgBiT-YAP-CC) and Small BiT with PPM1B (SmBiT-PPM1B) in lentiviral vectors to establish stable HeLa cells simultaneously expressing LgBiT-YAP-CC and SmBiT-PPM1B (Fig. S1A). The interaction between these proteins brings the two subunits into proximity to form a functional enzyme capable of producing a bright and luminescent signal. After ranking, we identified five compounds that markedly suppressed the luminescence intensity (fold change < 0.7) (Figs. 1B and S1B). Among these five compounds, paeoniflorin, verteporfin, and butoconazole nitrate significantly reduced TGF-β1-induced Smad binding element luciferase activity in human aortic smooth muscle cells (HASMCs) (Fig. 1C). Considering the cytotoxic effect of verteporfin [7] and the possible development of resistance by fungal organisms due to long-term butoconazole nitrate use [8], we focused on paeoniflorin, a major constituent of a Chinese herb [9]. We found that paeoniflorin showed no significant cytotoxicity (Fig. 1D) and inhibited the YAP-PPM1B interaction at a median inhibitory concentration (IC_50_) of 4.797 μM (Fig. 1E). Biolayer interferometry (BLI) experiments demonstrated a binding between paeoniflorin and the YAP-CC domain with an affinity of 4.05e × 10^−5^ M (Fig. 1F). Moreover, surface plasmon resonance analysis of PPM1B proteins covalently immobilized on a CM5 sensor chip, which was first saturated with YAP-CC domain peptide before the addition of paeoniflorin, revealed the inhibitory effect of paeoniflorin on the interaction between peptide and PPM1B (Fig. 1G). Immunoprecipitation analysis showed that paeoniflorin significantly reduced the YAP-PPM1B interaction (Fig. 1H), suggesting that paeoniflorin inhibited the PPM1B-YAP interaction. As a result, paeoniflorin reversed the ability of YAP to amplify TGF-β1-induced p-SMAD2/3 production in HASMCs (Fig. 1I and 1J).

**Figure 1.**
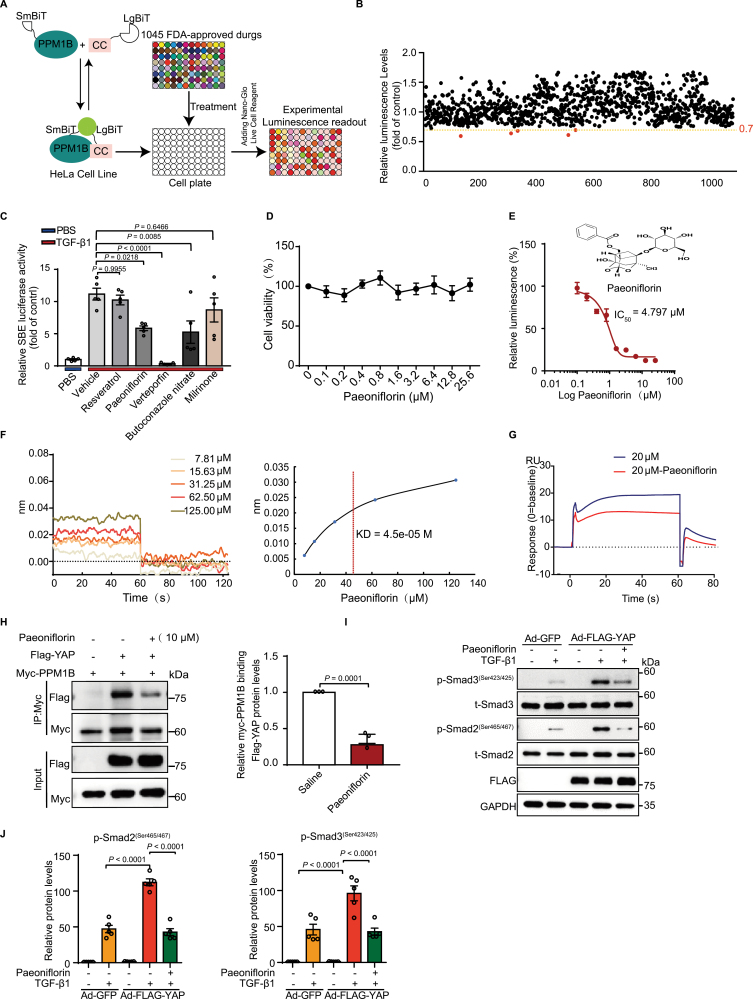
Paeoniflorin inhibits the binding of YAP to PPM1B. (A) HeLa cell line stably expressing both Flag-YAP-CC-LgBiT and Flag-PPM1B-SmBiT was plated in white 96-well cell culture plates and incubated for 12 h before treatment with 1045 FDA-approved drug screening library compounds (1 μM) or vehicle for 1 h. (B) The luminescence level relative to the vehicle is shown (each black dot represents one compound, and the compound ID is shown on the *x*-axis). The five compounds associated with a 0.7-fold change are circled in red (*n* = 3). (C) HASMCs were co-transfected with SBE luciferase reporter and β-galactosidase for 12 h and then incubated with the indicated compounds (10 μM) for 12 h before treatment with TGF-β1 (5 ng/mL) for another 6 h. SBE luciferase reporter activity was measured. *P*-values correspond to a two-way analysis of variance (ANOVA) with Bonferroni post-test (*n* = 5). (D) HeLa cell lines stably expressing both YAP-CC-LgBiT and PPM1B- SmBiT were plated in white 96-well cell culture plates for 12 h and then treated with the indicated concentration gradient of paeoniflorin for 12 h. Cell-counting kit-8 solution was then added, and the cells were incubated for 4 h. Luminescence was then monitored at 450 nm (*n* = 8). (E) HeLa cell lines stably expressing LgBiT-YAP-CC and SmBiT-PPM1B were incubated in 96-well white cell culture plates for 12 h and then treated with different concentrations of paeoniflorin (10 μM) for 1 h to monitored their luminescence. *P*-values correspond to one-way ANOVA with Bonferroni post-test (*n* = 3). (F) BLI reaction analysis of the binding and dissociation of Yap-cc from paeoniflorin. (G) Surface plasmon resonance analysis of the competitive binding of peptide (SNSNQQQQMRLQQLQMEKERLRLKQQELLRQ) and paeoniflorin with PPM1B. (H) HEK293T cells were co-transfected with Myc-PPM1B and FLAG-YAP for 48 h before treatment with paeoniflorin (10 μM) for 1 h. *P*-values correspond to unpaired two-tailed *t*-tests (*n* = 3). (I–J) HASMCs were infected with Ad-GFP or Ad-FLAG-YAP for 48 h before incubation with paeoniflorin (10 μM) for 12 h followed by treatment with TGF-β1 (5 ng/mL) for 1 h. (I) Western blot analysis of p-Smad2(ser465/467), p-Smad3(ser423/425) and total Smad2/3 protein levels. (J) Quantitative analysis of p-Smad2ser465/467 (left panel) and p-Smad3ser423/425 (right panel) protein levels. *P*-values correspond to two-way ANOVA with Bonferroni post-test (*n* = 5).

We then assessed the therapeutic effects of paeoniflorin on HFHS-induced arterial stiffness in mice. Treatment with paeoniflorin did not affect the body weight, the epididymal adipose mass to body weight ratio, plasma total cholesterol and triglyceride levels, and glucose tolerance (Fig. S1C and S1D). Paeoniflorin significantly relieved the arterial stiffness in mice fed an HFHS diet for 8 weeks, evidenced by increased circumferential cyclic strain and decreased pulse wave velocity (PWV) of the aorta and left carotid artery (LCA), which were unchanged in mice receiving a normal diet (ND) (Fig. 2A). Similarly, morphologic and immunofluorescence analyses showed that paeoniflorin reduced collagen accumulation (Fig. 2B and 2C) in the aorta and carotid arteries of HFHS-fed mice fed. Immunofluorescence analysis revealed increased co-localization of YAP and PPM1B in the aortic tunica media of HFHS-fed mice, which was abolished by paeoniflorin (Figs. 2D and S1E). Moreover, the p-Smad2/3 protein levels were reduced by paeoniflorin in the tunica media of HFHS-fed mice whereas those of YAP were unchanged (Figs. 2E,F and S1F,H).

**Figure 2.**
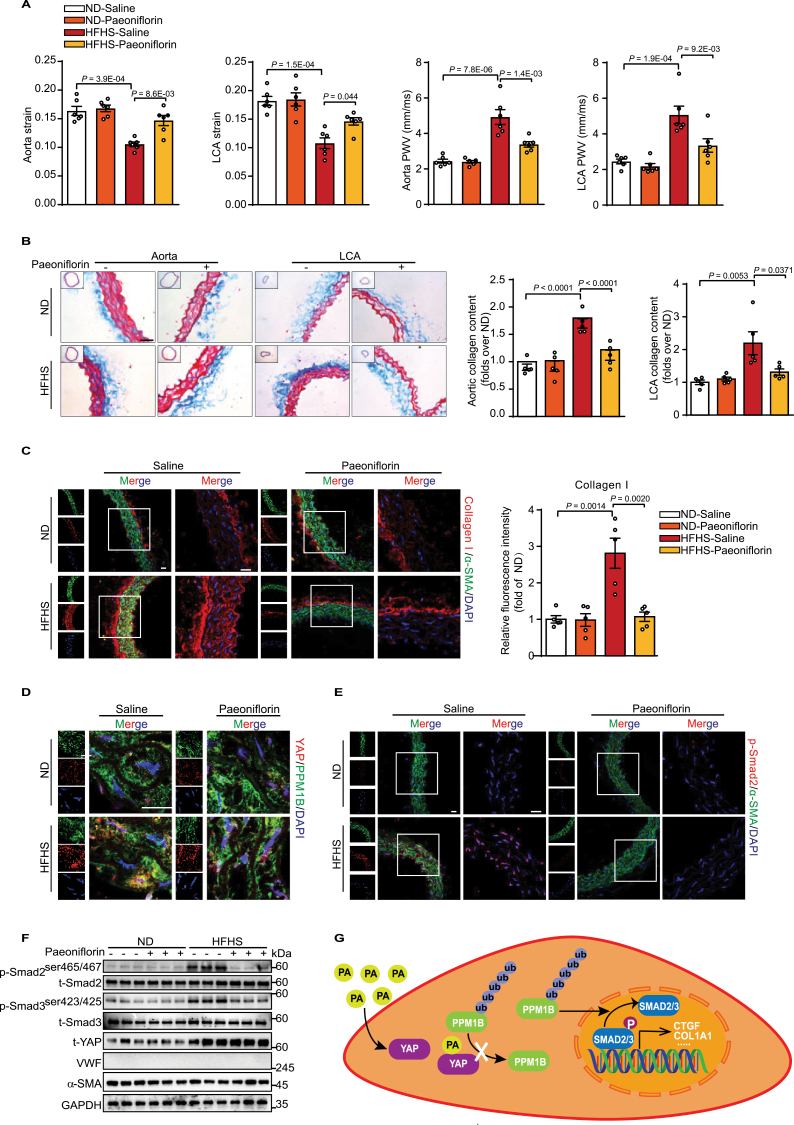
Paeoniflorin relieves HFHS-induced arterial stiffness. (A–G) C57BL/6J mice (aged 8 weeks) were fed a HFHS diet or a ND and intraperitoneally injected with saline or paeoniflorin (100 mg/kg/day) for 8 weeks. (A) Quantification of the circumferential cyclic strain and PWV of the aorta and carotid artery of mice (*n* = 6). (B) Representative images of Masson’s trichrome staining of the aorta and LCA of mice. Scale bars: 20 μm. Quantitative analysis of collagen content in the aorta and carotid artery based on Masson’s trichrome staining (*n* = 5). (C) Immunofluorescence staining of collagen I (red), α-SMA (green), and DAPI (blue) in cross-sections of mouse aorta (*n* = 5). Scale bars: 20 μm. Quantitative analysis of collagen content in the aorta and carotid artery based on immunofluorescence staining (*n* = 5). (D) Immunofluorescence staining of YAP (red), PPM1B (green), and DAPI (blue) in cross-sections of mouse aorta (*n* = 5). Scale bars: 20 μm. (E) Immunofluorescence staining of p-Smad2 (red), α-SMA (green), and DAPI (blue) in cross-sections of mouse aorta (*n* = 5). Scale bars: 20 μm. (F) Western blot analysis of p-Smad2^(ser465/467)^, p-Smad3^(ser423/425)^, total Smad2/3, α-SMA and total YAP protein levels in the aortic tunica media of mice (*n* = 5). (G) Schematic summary the mechanism of paeoniflorin in inhibiting arterial stiffness. In all analyses, *P*-values correspond to two-way ANOVA with Bonferroni post-test.

Taken together, these findings indicate that paeoniflorin attenuates HFHS-induced arterial stiffness and abnormal extracellular matrix accumulation by disrupting the interaction of YAP and PPM1B and subsequently inhibiting the prolonged activation of the TGF-β pathway.

## Research limitations

Our research describes that paeoniflorin can prevent the development of arterial stiffness by inhibiting the binding of PPM1B to YAP, providing new insights into the treatment of arterial stiffness. Although we found that paeoniflorin binds to the YAP-CC structure, we did not investigate whether paeoniflorin directly acts on PPM1B.

## Supplementary Material

lnad029_suppl_Supplementary_Material
