# Supplementary material for: Paeoniflorin relieves arterial stiffness induced by a high-fat/high-sugar diet by disrupting the YAP-PPM1B interaction
Source: Life Med. 2023 Sep 12;2(5):lnad029. doi: 10.1093/lifemedi/lnad029 (PMC11749085; doi:10.1093/lifemedi/lnad029)

Supplementary Information

Paeoniflorin relieves arterial stiffness induced by a high-fat/high-sugar diet by disrupting the YAP-PPM1B interaction

Materials and methods

Animals

Eight-week-old male C57BL/6J mice were purchased from Beijing Vital River Laboratory Animal Technology (Beijing, China). All mice were maintained in SPF facility conditions before experiments. All animal experiments were conducted in accordance with the National Institutes of Health Guide for the Care and Use of Laboratory Animals and approved by the Institutional Animal Care and Use Committee of Tianjin Medical University. Mice were fed a high-fat, high-sucrose diet or a normal diet.

Glucose tolerance test (GTT)

After 8 hours of fasting, mice were injected with 30% glucose at a dose of 2g/kg body weight and then their blood glucose was measured at 0, 15, 30, 60, 90, 120 minutes using a blood glucose meter.

Immunoprecipitation

The cells are lysed using lysate and their whole cell lysate is extracted. To quantify the protein concentration using BCA, 1 mg of protein was added to 20 uL of anti-myc immunomagnetic beads overnight at 4°C and then the beads were washed 5 times with buffer (NaCl 136.89 mM, KCl 2.67 mM, Na_2HPO_4 8.1 mM, KH_2PO_4 1.76 mM, and 0.5% Tween20). After the last wash the supernatant was removed leaving the immunomagnetic beads, which were resuspended by adding 1X SDS and then boiled for 10 minutes followed by western blot analysis.

Immunofluorescence staining

Arterial sections were incubated in PBS buffer containing 5% goat serum, 0.1% Triton X-100 for 1 hour at room temperature, followed by incubation of the primary antibody

corresponding to the protein overnight at 4°C, followed by two washes with PBS and incubation of the secondary antibody for one hour at room temperature. The ratios of antibodies used were: anti-collagen type I (1:200), anti-a-SMA (1:100), anti-YAP (1:100), anti-p-Smad2$^{ser465\ /Ser467}$ (1:80), anti-PPM1B (1:80). Fluoroshield mounting medium with DAPI was used to cover slides, and images were captured using a Zeiss confocal laser-scanning microscope.

Nanoluc Binary Technology (Nanobit)-based binding assay

The LgBiT and SmBiT genes were inserted at the C-terminus of the YAP-CC domain or PPM1B to obtain the lentiviral constructs encoding YAP-CC-LgBiT and PPM1B-SmBiT, respectively. A stable HeLa cell line expressing both YAP-CC-LgBiT and PPM1B- SmBiT was established. The cells were seeded into white 96-well cell culture plates. Nano-Glo live cell reagent was added to each well and allowed to incubate for 10 min at 37°C, and the baseline luminescence was measured. Diluted compounds from the FDA-approved drug screening library or vehicle at a final concentration of 10 μM were added, and luminescence was monitored after 1 h. The luminescence levels were nomalized to the baseline value, and the compounds with relative luminescence level below 0.7 were selected.

Vascular stiffness

Aortic stiffness was assessed by measuring pulse wave velocity and circumferential cyclic strain in mice using the ultra-high resolution small animal ultrasound imaging system vevo2100, recording aortic pulse waves in B-mode and aortic diameter and vessel wall thickness in M-mode. Systolic diameter (Ds) and diastolic diameter (Dd) were recorded and circumferential cyclic strain (Ds – Dd)/Dd was calculated. Pulse travel time was calculated as the transit time of each waveform between the sites using the R-wave of the ECG as a reference point. PWV (mm/ms) was calculated by dividing the distance by transit time.

Cell culture and plasmid transfection

HEK293T cells and Hela cells were cultured in DMEM medium containing 10% fetal bovine serum and 1% penicillin-streptomycin solution. 0.05 mg/mL ascorbic acid; 0.01 mg/mL insulin; 0.01 mg/mL transferrin; 10 ng/mL sodium selenite; 0.03 mg/mL

endothelial cell growth supplement (ECGS); 10 mM HEPES, 10 mM TES and 10% fetal bovine serum and 1% penicillin-streptomycin were added to the basal F-12 medium,Human aortic smooth muscle cells were then cultured in the medium. HEK293T cells were grown to 70%–80% when plasmid transfection was performed using TransIntro® EL Transfection Reagent.

Luciferase reporter assay

HASMCs cells were transfected with the Smad binding element (SBE) luciferase reporter and β-galactosidase for 24 h, and then incubated with TGF-β1 (5 ng/mL) for 6 h. Luciferase activities were measured using a luciferase assay system (Promega, Madison, WI) and β-galactosidase was quantified using the enzyme assay system as an internal control (Promega, Madison, WI).

Extraction proteins and western blot analysis

Animal tissue or cell lysates using RIPA lysates which contain complete protease-inhibitor cocktail (Cat. No. 04693132001; Roche, Indianapolis, IN, USA), phosphatase inhibitors (Cat. No. 04906845001; Roche) and PMSF (Cat. No. P0100; Solarbio Life Sciences; Beijing, China). For western-blot analysis, protein samples were separated by SDS-PAGE and transferred onto nitrocellulose membranes. Nitrocellulose membranes are incubated with primary antibody after closure. Rabbit anti-YAP (Cat# 14074S), Rabbit anti-p-Smad2 $^{(Ser465/467)}$ (Cat# 3108), Rabbit anti-Smad2 (Cat#5339) Rabbit anti-Smad3 (Cat#9523) Rabbit anti-DYKDDDDK Tag (Cat#14793) were purchased from Cell Signaling Technology, Rabbit anti-p-Smad3 $^{(Ser423/425)}$ (Cat# ab52903) and Rabbit anti-VWF (Cat# ab154193) were purchased from Abcam, Mouse anti-GAPDH (Cat#60004-1-Ig) was purchased from ProteinTech, Mouse anti-DYKDDDDK Tag (Cat# F1804) were purchased from Sigma-Aldrich, Rabbit Anti-α-SMA (Cat#14-9760-82) was purchased from ThermoFisher. Quantification of band intensity of immunoblots using ImageJ software.

Surface Plasmon Resonance (SPR) experiment

The interactions between peptides and proteins were investigated using the Biacore 8 K system (Cytiva, USA). The recombinant PPM1B protein was diluted in 10 mM acetate (pH 5.0) and immobilized on a Biacore CM5 sensor chip via primary amine

groups. Approximately 15000 resonance units (RU) of the immobilized protein were obtained. Interaction analyses were performed using HBS-P containing $MgCl_2$ (10 μM) as a running buffer. Increasing concentrations of polypeptides (SciLight Biotechnology) were passed through the chip at a flow rate of 30 μL/min, and data were analyzed using Biacore evaluation software (8k Version 2.0.15), and the curve was fitted with a 1:1 binding model. To determine competition with the compounds, PPM1B was immobilized on a CM5 sensor chip. The polypeptide (20 μM) or mixed with compound (20 μM) was injected onto the chip until a binding steady state was reached.

Biolayer interferometry (BLI) experiment

The binding affinity of Paeoniflorin to YAP-CC was determined using the Octet Red 96 Molecular Interaction Analyser. The His-YAP-CC protein was coupled to the NI-NTA biosensor, and for each assay, five NI-NTA biosensors were taken and placed on the sensor tray and soaked in PBST buffer for 10 min. The His-YAP-CC protein was diluted to 100 ug/mL with PBST protein solution, and the prepared Paeoniflorin PA was diluted with PBST to form five concentration gradients, and finally the His-YAP-CC protein, and the gradient diluted PA, buffer were added to the sample plate for detection. A 1:1 binding model was assumed in binding kinetics analysis. KD, Kon, Koff and R2 values were reported.

Figure S1. Paeoniflorin relieved HFHS-induced arterial stiffness by disrupting YAP-PPM1B interaction.

(**A**) Immunoblot analysis of Flag-YAP-CC-LgBiT and Flag-PPM1B-SmBiT protein levels in stably transfected HeLa cell lines using an anti-FLAG antibody. (**B**) HeLa cell lines stably expressing LgBiT-YAP-CC and SmBiT-PPM1B were plated in white 96-well cell culture plates for 12 h, and then treated with the indicated compounds (1 μM) for 1 h before luminescence was monitored. *P*-values correspond to one-way ANOVA with Bonferroni post-test (n = 3). **(C)** Plasma levels of glucose were measured after intraperitoneal injection of 30% glucose to achieve a final dose of 2 mg/kg body weight (n = 5). **(D)** The epididymis adipose mass (EAM)/body weight ratio of mice (n = 10). Mice were fasted for 12 h before total plasma cholesterol (CHO), triglycerides (TG), high-density lipoprotein cholesterol (HDL-C) and low-density lipoprotein cholesterol (LDL-C) levels were measured. n = 10. **(E)** Quantitative analysis fluorescence intensities of YAP co-localization with PPM1B in Fig. 2D (n = 5). **(F)** Quantitative analysis fluorescence intensities of p-Smad2 in cross-sections of mouse aorta of Fig. 2E (n = 5). **(G)** Quantitative analysis of total YAP protein , p-Smad2(ser465/467) and p-Smad3(ser423/425) protein levels in the aortic tunica media of mice (n = 5). **(H)** Immunofluorescence staining of YAP (red), a-SMA (green) and DAPI (blue) in cross-sections of mouse aorta (n = 5). Scale bars: 20 μm. Quantitative analysis of YAP in cross-sections of mouse aorta based on immunofluorescence staining (n = 5).

Supplemental

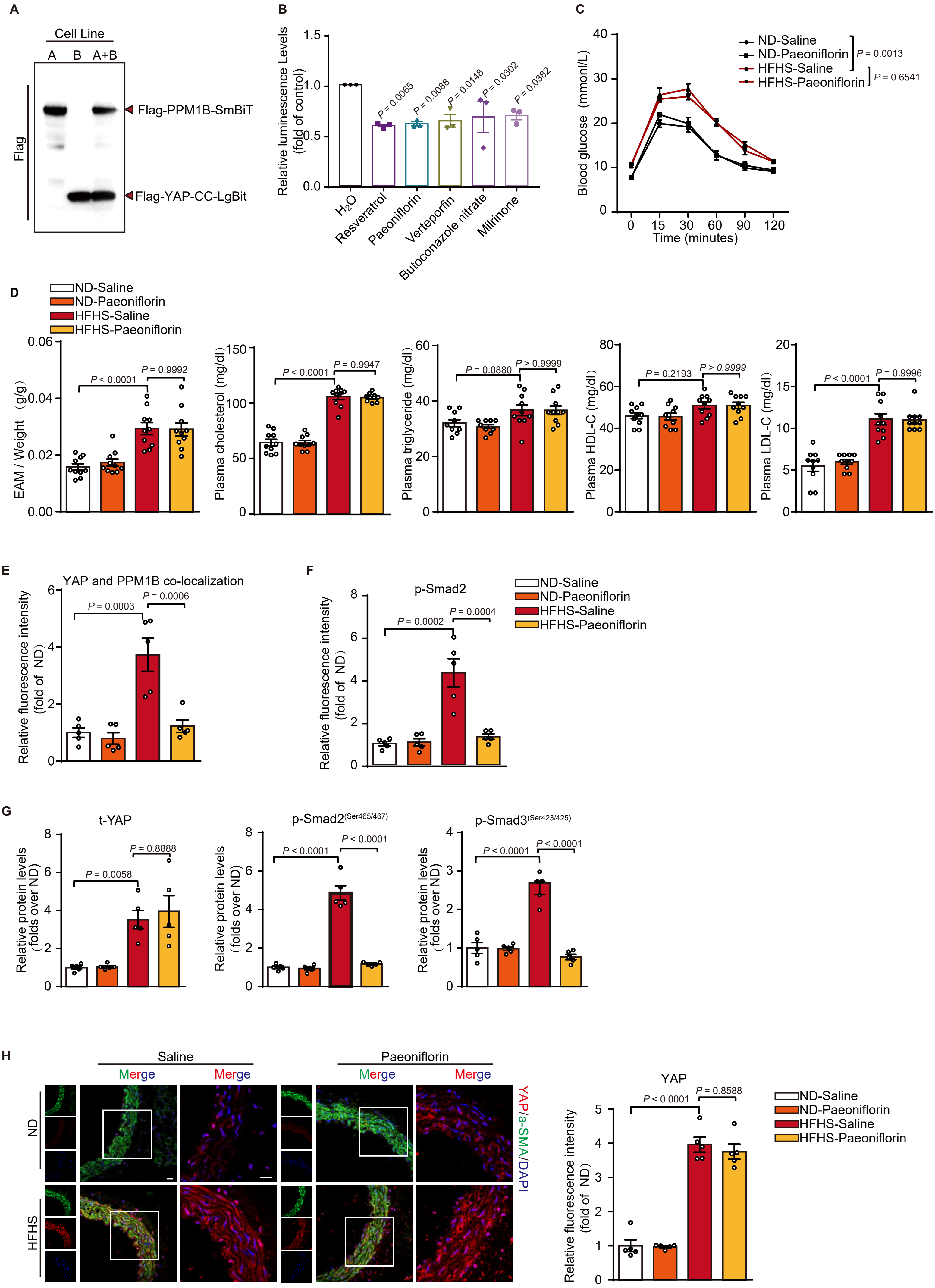

Supplement: lnad029_suppl_Supplementary_Material [file lnad029_suppl_Supplementary_Material.pdf]
